# Book Review

**Published:** 1909-06

**Authors:** 


**A Practical Course of General Physiology.** By D. NOEL PATON, M.D., and G. HERBERT CLARK, M.B. Pp. 39. Glasgow: James Maclehose & Sons. 1908.—This is a convenient syllabus for a practical class in physiology. It proceeds on the novel plan of giving none of the deductions from the experiments described. The student is expected to fill that in for himself, and the book is interleaved for the purpose. This should help him to remember the work. One is glad to see that the medical student is not burdened with too much detail about the reactions of nerve-muscle preparations, and that adequate attention is given to the special senses. The important experiments on these are too often neglected.

**Encyclopædia and Dictionary of Medicine and Surgery.** Vol. VII, *Ner–phy*. Pp. xii, 579. Edinburgh: William Green & Sons. 1908.—The chief articles in this volume are on "Diseases of the nerves, the nose, eye, pancreas, peritoneum, and pharynx." Of the excellence of the articles as a whole and their general arrangement we can speak with confidence, the majority being by leading authorities in their several specialities. It is a matter for some regret, however, that these authors have not always had the opportunity of bringing their contributions up to date. For instance, for deviations of the septum without ridges we are told that the only satisfactory operation is that of Dr. Asche, of New York, no mention being made of the operation now almost universally practised—that of "submucous resection."

**Guide to the Clinical Examination and Treatment of Sick Children.** By John Thomson, M.D. Second edition. Pp. xxviii, 629. Edinburgh: William Green & Sons. 1908.—This book, as it now appears in a revised and enlarged form, is one of the best expositions of pædiatrics in the English language. Set descriptions of morbid anatomy are purposely left out; but the clinical aspect of disease in children is expounded in a masterly manner. Everyone interested in the disorders of childhood should read this book, not only for the sake of its intrinsic value, but also because it is an inspiring example of the fruitfulness of industry linked with freedom from prejudice. The book is well printed on rather heavy paper, and its value is greatly enhanced by the illustrations, almost all of them reproduced photographs.

**Common Affections of the Liver.** By W. Hale White, M.D. Pp. viii, 302. London: James Nisbet & Co. Ltd. 1908. —The author apologises for the appearance of another book on the liver, but the clinical teaching contained herein is of the highest value, and is presented in an attractive form, such as will appeal to students beginning their work, rather than those who have extensive clinical knowledge. The author has little sympathy with those patients who call everything "liver." He remarks that there is no evidence that the symptoms so-called are due to liver at all. Dr. Hale White is a clinical teacher of large experience, and his views on all the commonly-accepted diseases of this organ must command universal esteem.

**Essentials of Surgery.** By Alwyne T. Compton, F.R.C.S. Pp. viii, 428. London: Henry Kimpton. 1908.—The author has evidently expended much time and pains in the compilation of this book, which is above the average of its class in merit and arrangement, is in legible type, has numerous good illustrations, and is well indexed. There are several clerical mistakes, and the information—as with fractures of the lower end of the humerus—is not always up to date. Nevertheless, we can commend the work as a suitable one for those who wish to revive their memories before examination, and for occasional reference by those with a limited affection for more comprehensive literature.

**Golden Rules of Venereal Disease.** By C. F. Marshall, M.D., F.R.C.S. Pp. 90. Bristol: John Wright & Co. n.d.—This is one of the waistcoat-pocket size booklets of the well-known "Golden Rules" Series, and deals with syphilis, gonorrhœa, and soft chancre. The author, whose larger work on the same subject we have had occasion to praise highly in a former number of our *Journal*, here provides the reader with an excellent epitome of the leading points in the pathology, symptomatology and treatment of the diseases mentioned. Each "golden rule" emphasises some important matter which the tyro, at least, might in practice forget. We consider that the space at the writer's disposal has been very well used.

**"The Ophthalmoscope."** Vol. VI, No. 4. Pp. 114 London: George Pulman & Sons.—This number of the *Journal* is devoted almost entirely to articles on disease of the eye in relation to accessory sinus disease of the nose. From cover to cover the matter is full of interest, and forms a fairly complete *résumè* of present-day knowledge of these important questions. The editors are to be congratulated on their progressive foresight and literary enterprise

**Hay Fever, Hay Asthma: its Causes, Diagnosis and Treatment.** By William Lloyd. Second Edition. Pp. 101. London: H. J. Glaisher. 1908.—This short work on a very prevalent disease reviews various investigations that have been made from time to time, and some of the theories that have been put forth to account for the symptoms, and details methods of treatment that have been adopted by laryngologists and physicians. The author states in his preface that he "does not delude himself with the idea that this little work will supply a 'long-felt want.' The chief reason for writing it is to record certain opinions that the author holds upon causes and effective treatment of hay fever." It is not very clear to us, on reading the work, what are the distinctive views which are the main object in producing the work, although we see that he accepts the classification which differentiates the varieties of pseudo-hay fever into the sensori-motor and ideo-motor forms—a classification which, as far as we are aware, has only been given once before. We are glad, however, to see that he condemns in no measured terms the curious advice of "one author," that in obstinate cases the complete removal of middle and inferior turbinates is a method to be adopted. As regards the author's own series of cases, it is refreshing to find that touching with the cautery produces in so many cases the "result, cure." We only wish a little further detail were given, as temporary cures are as prolific as final failures in the hands of most practitioners. It is difficult to see in what respects this work adds to our knowledge of this affection, but that it meets a need is shown by the fact that a second edition has already been called for.

**The Law in General Practice.** By STANLEY B. ATKINSON, M.A., M.B., B.Sc. Pp. viii, 239. London: Henry Frowde and Hodder and Stoughton. 1908.—Mr. Atkinson is well qualified to write upon this subject, and we can thoroughly recommend his book to all medical men commencing, or not fully experienced in practice. It deals with a large number of contingencies in matters forensic, ethical and medical, and abounds with useful hints and profitable warnings. The book is easily readable, and if well digested should prove of the utmost value to the young professional man, in affording him a ready-made experience and helpful counsel in dealing with many perplexing occasions almost certain to beset his early career.

**The Care and Nursing of the Insane.** By PERCY J. BAILEY, M.B., C.M. Pp. 270. London: The Scientific Press.—This little book is written for candidates for the nursing certificate of the Medico-Psychological Association. It gives an outline of anatomy and physiology, it indicates some of the chief symptoms of disease, both physical and mental, and their significance in such a manner that they may be understood by the uninitiated. We think the author has succeeded in his endeavours. The book should be useful to those for whom it is designed.

**Death and its Verification.** By J. BRINDLEY JAMES. Pp. 56. London: Rebman Limited. 1908.—The author of this booklet is the examiner-in-chief for the Association for the Prevention of Premature Burial. He gives fourteen different tests, including the injection of fluorescin and an X-ray examination of the heart. The use of the stiletto, or still better an efficient *post-mortem* examination, must effectually prevent burial before death. The death verification bag, with its thirteen weapons, can rarely be necessary to satisfy any medical man as to the fact of death. The usual armamentarium of the doctor's pocket should suffice.

**The Extra Pharmacopœia of Martindale and Westcott.** Revised by W. Harrison Martindale, F.C.S., and W. Wynn Westcott, M.B. Thirteenth Edition. Pp. xl, 1164. London: H. K. Lewis. 1908.—A thinner volume than the last, but containing nearly 200 pages more. This has been accomplished by the use of a different paper, and by resetting in smaller type a large amount of the matter to which reference is less frequently made. Both prescriber and dispenser must find the book to be an invaluable and reliable guide. Everything new will be found there, but the print is now so small that a magnifying glass becomes essential to the reader.

**Index-Catalogue of the Library of the Surgeon-General's Office, United States Army.** Second Series. Vol. XIII. Pp. 929. Washington: Government Printing Office. 1908.—The present volume includes 5,566 author titles, 7,678 subject-titles of separate books and pamphlets, and 40,221 titles of articles in periodicals. No praise is too high for the painstaking labour involved in the preparation of this most valuable and necessary work of reference, which is a model of completeness and accuracy. As an example of its extensive character, we may mention the subject of "Plague," to which fifty-three pages of references are given. "Pregnancy" similarly occupies more than a hundred pages.

**A Practical Text-Book of Infectious Diseases.** By R. W. Marsden, M.D. Pp. vi, 296. Manchester: University Press. 1908.—When so capable and well-known a writer as Dr. Marsden undertakes a book which is to fill a real gap in the ranks of medical literature, it would seem that all the essentials of a successful venture are found. Unfortunately the actual fruit of his labours does not quite fulfil these expectations. The book is a compromise. On the one hand, it is not a complete and exhaustive treatise, indeed, the author assures us that he did not mean it to be. Yet he has not, on the other hand, realised the alternative plan which he set before himself, for he has written a book in which it is not easy to refer quickly to any special point. We think it would be better in future editions to condense the subject-matter, and arrange it under headings so printed as to catch the eye readily. If this could be done, there is no doubt that the book would find adequate support from medical men, and probably from students also. Some illustrations would make it more interesting.

